# Transcription factor TFEB cell-autonomously modulates susceptibility to intestinal epithelial cell injury *in vivo*

**DOI:** 10.1038/s41598-017-14370-4

**Published:** 2017-10-24

**Authors:** Tatsuro Murano, Mehran Najibi, Geraldine L. C. Paulus, Fatemeh Adiliaghdam, Aida Valencia-Guerrero, Martin Selig, Xiaofei Wang, Kate Jeffrey, Ramnik J. Xavier, Kara G. Lassen, Javier E. Irazoqui

**Affiliations:** 1 Gastrointestinal Unit and Center for the Study of Inflammatory Bowel Disease, Massachusetts General Hospital, Harvard Medical School, Boston, MA 02114 USA; 2 Department of Pathology, University of Massachusetts Medical School, Worcester, MA 01605 USA; 3 The Broad Institute of MIT and Harvard, Cambridge, MA 02142 USA; 4 Department of Pathology, Massachusetts General Hospital, Harvard Medical School, Boston, MA 02114 USA; 5 Present Address: Department of Microbiology and Physiological Systems, University of Massachusetts Medical School, Worcester, MA 01605 USA

## Abstract

Understanding the transcription factors that modulate epithelial resistance to injury is necessary for understanding intestinal homeostasis and injury repair processes. Recently, transcription factor EB (TFEB) was implicated in expression of autophagy and host defense genes in nematodes and mammalian cells. However, the *in vivo* roles of TFEB in the mammalian intestinal epithelium were not known. Here, we used mice with a conditional deletion of *Tfeb* in the intestinal epithelium (*Tfeb* ^ΔIEC^) to examine its importance in defense against injury. Unperturbed *Tfeb* ^ΔIEC^ mice exhibited grossly normal intestinal epithelia, except for a defect in Paneth cell granules. *Tfeb* ^ΔIEC^ mice exhibited lower levels of lipoprotein *ApoA1* expression, which is downregulated in Crohn’s disease patients and causally linked to colitis susceptibility. Upon environmental epithelial injury using dextran sodium sulfate (DSS), *Tfeb* ^ΔIEC^ mice exhibited exaggerated colitis. Thus, our study reveals that TFEB is critical for resistance to intestinal epithelial cell injury, potentially mediated by APOA1.

## Introduction

The intestinal epithelium is the major site of interaction between the host and colonizing microbes. It is formed by one layer of intestinal epithelial cells, which include enterocytes, mucus-secreting goblet cells, antimicrobial-secreting Paneth cells, and hormone-secreting enteroendocrine (or chromaffin) cells. A major function is to provide a physical barrier to bacterial translocation into the host. An equally important function of intestinal epithelial cells is to secrete antimicrobial peptides and mucus that establish spatial segregation with the microbial population that resides in the lumen. A third important function is of surveillance of the lumen. Disruption of homeostasis in the lumen, by bacterial dysbiosis or chemical insult, results in the induction of genes that encode signaling molecules, which are secreted into the underlying stroma and recruit cells of the innate and adaptive immune systems^1,2^. Defective epithelial barrier function results in chronic activation of such responses, leading to inflammatory bowel disease and malignancy^3^.

Intestinal epithelial cells sense damage to the epithelial barrier as well as microbial products, which triggers changes in gene expression resulting in activation of the downstream host response to restore homeostasis^4,5^. Although the regulation of intestinal epithelial gene expression by such stimuli is considered a key event to preserve and restore homeostasis, it is poorly understood. Previous studies have found roles for the NF-κB family of transcription factors that are triggered by engagement of TLR and NLR pattern recognition receptors^2,6–8^. However, it is likely that more complex transcriptional networks are important for the regulation of gene expression in the intestinal epithelium in response to environmental cues^9,10^.

To discover transcription factors that are important for host defense in the intestinal epithelium, we previously followed an unbiased approach in the model organism *C*. *elegans*. We discovered that transcription factor TFEB (called HLH-30 in nematodes) is important for the induction of host defense genes in *C*. *elegans* infected with pathogenic bacteria^11^. TFEB was previously discovered as a transcription factor binding to the Eµ heavy chain immunoglobulin enhancer^12^, and important for the induction of CD40 ligand in T cells^13^. More recently, TFEB was identified as a key transcriptional activator of a large set of genes during cellular stress, including autophagy and lysosomal genes, known as the Coordinated Lysosomal Expression and Regulation (CLEAR) network^14–16^.

TFEB activity is regulated post-translationally. Phosphorylation of TFEB by mTORC1, ERK2, and GSK3 results in its cytosolic retention^16–18^. Dephosphorylation of TFEB can be triggered by release of lysosomal Ca^**2+**^ and subsequent activation of protein phosphatase calcineurin, as well as by mTOR inhibition, causing its relocalization into the nucleus^19^. In our previous studies, we discovered that *C*. *elegans* HLH-30/TFEB controls the induction of genes with antimicrobial or cytoprotective functions, and that both classes of genes are required for host defense against infection. However, whether HLH-30/TFEB is required within the intestinal epithelium for this function is not known. We also showed that TFEB is required in murine macrophages for proper induction of several cytokines and chemokines after phagocytosis of Gram+ or Gram- bacterial pathogens, indicating that TFEB is a novel and evolutionarily conserved transcription factor in the host response to bacterial infection^11,20^. Based on this precedent, it is important to determine the functional importance of TFEB in barrier protection and repair in the mammalian intestinal epithelium.

To examine the biological roles of intestinal epithelial TFEB *in vivo*, we generated mice that lack intestinal epithelial TFEB. Conditional deletion of TFEB in the intestinal epithelium did not cause major abnormalities in ileal or colonic epithelium in unperturbed animals, except for abnormal Paneth cell secretory granules. In contrast, deletion of TFEB resulted in dramatically enhanced pathology during DSS-induced colitis, including increased local and systemic inflammation and bacterial translocation to distal tissues, concomitant with large gene expression changes in the intestinal epithelium compared to wild type animals. These strong effects of TFEB deletion reveal an important and previously unknown function for TFEB in resistance to injury to the intestinal epithelium.

## Results

### Intestinal epithelial TFEB is dispensable for baseline homeostasis

**Figure 1:**
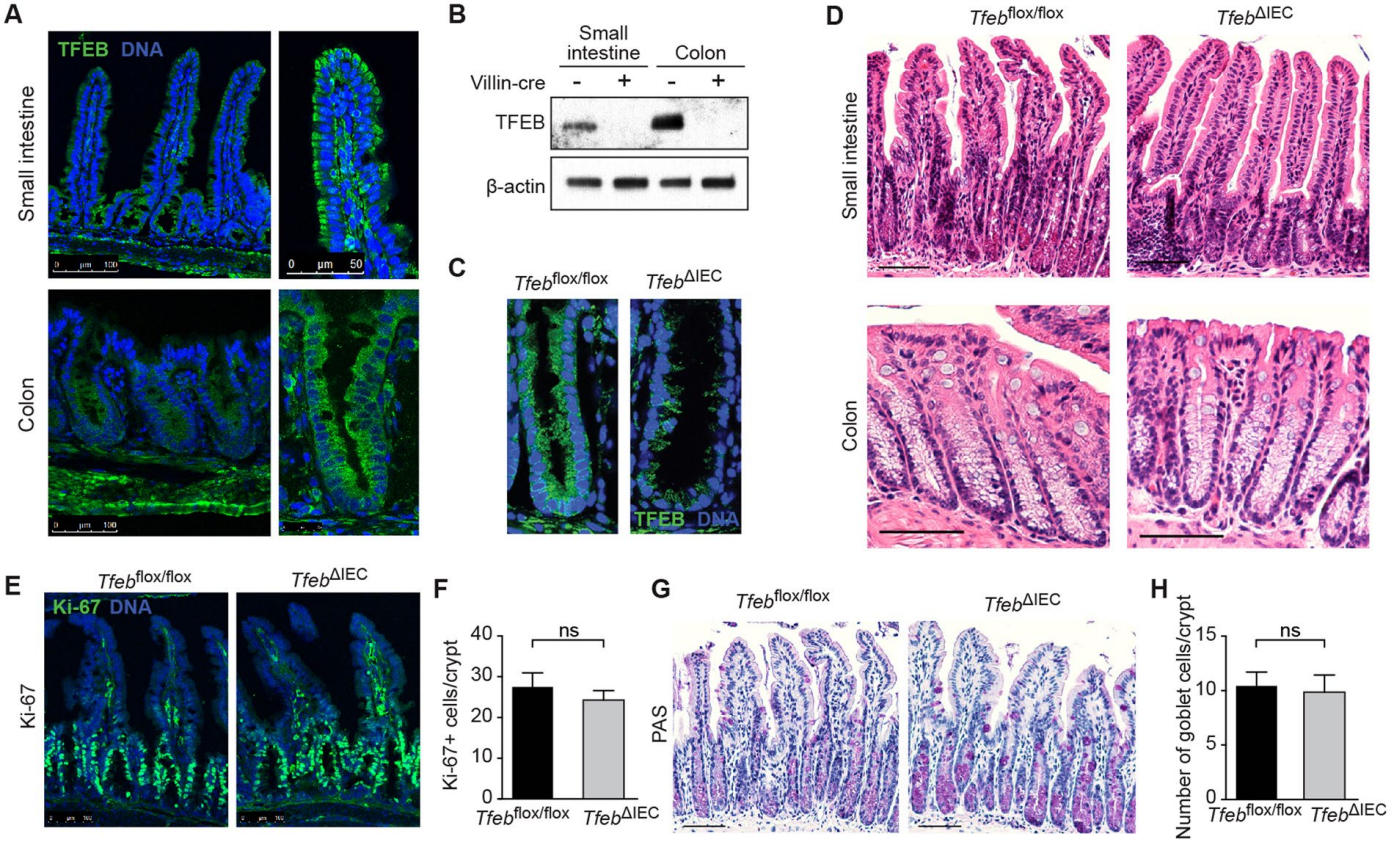
*Tfeb* ^ΔIEC^ animals exhibit normal gut morphology. (**A**) Anti-TFEB immunofluorescence of small intestine (*Top*) or colon (*Bottom*). (**B**) Anti-TFEB immunoblot of whole cell extracts showing undetectable levels of TFEB in the enterocytes of *Tfeb* ^ΔIEC^ animals. (**C**) Anti-TFEB immunofluorescence of colonic crypts. (**D**) H&E staining of sections of small intestine (*Top*) and colon (*Bottom*) of *Tfeb* ^ΔIEC^ and *Tfeb* ^*flox/flox*^ animals. Scale bars represent 100 μm. (**E)** Anti-Ki-67 immunofluorescence of small intestine of *Tfeb* ^ΔIEC^ and *Tfeb* ^*flox/flox*^ animals. Scale bars represent 100 μm. (**F**) Quantification of Ki-67 immunofluorescence. N = 4 each. Data are means, error bars are S.E.M. ns, not significant (Student’s t-test). (**G)** PAS staining of small intestine sections of *Tfeb* ^ΔIEC^ and *Tfeb* ^*flox/flox*^ animals. Scale bars represent 100 μm. (**H)** Quantification of goblet cells per ileal crypt. N = 3 mice per genotype and at least 20 crytps were scored for each mouse. Data are means, error bars are S.E.M. ns, not significant (Student’s *t* test).

To determine the pattern of expression of TFEB in unperturbed animals, we performed anti-TFEB immunofluorescence in sections of the small intestine and colon of 8–12 week old mice of both sexes. We observed extensive TFEB expression throughout both tissues (Fig. 1A). In the small intestine, TFEB expression was prominent in the epithelium proper along the crypt-villus axis, with higher expression in the crypts. TFEB was also expressed by unidentified cells in the lamina propria. While TFEB expression was apparent in the submucosa and adventitia, it was absent from the muscularis propria. Similarly, in the colon TFEB expression was mostly epithelial, submucosal, and adventitial. Furthermore, qRT-PCR of *Tfeb* mRNA from colon revealed that *Tfeb* mRNA is enriched in the epithelial cells compared to whole tissue (Fig. S1). These observations show that in the intestine, TFEB is expressed mainly in the epithelium.

To investigate the biological importance of TFEB expression in the intestinal epithelium, we undertook loss of function studies. *Tfeb*-knockout mice exhibit embryonic lethality^21^. Therefore, to generate mice that lack intestinal epithelial TFEB expression, we bred *Tfeb* ^*flox/flox*^ mice with mice that express the site-specific recombinase Cre from the villin promoter (*villin-Cre* mice)^18^. The resulting *Tfeb* ^*flox/flox*^ homozygous, *villin-Cre* heterozygous double mutants (*Tfeb* ^ΔIEC^ mice) lack detectable TFEB expression in the intestinal epithelium (Fig. 1B,C). In terms of growth, size, weight, external appearance, and behavior, *Tfeb* ^ΔIEC^ mice were indistinguishable from their *Tfeb* ^*flox/flox*^ littermates. Assessment of small intestine and colon from *Tfeb* ^ΔIEC^ mice revealed normal histology compared to age-matched, *Tfeb* ^*flox/flox*^ littermates (Fig. 1D), regardless of sex. Furthermore, there were no obvious differences in cell proliferation in either small intestine (Fig. 1E,F) or colon (not shown), as measured by Ki-67 immunofluorescence of tissue sections, nor in goblet cell numbers, as measured using Periodic-acid Schiff (PAS) stain (Fig. 1G,H). Thus, we concluded that TFEB is dispensable for normal morphology, proliferation, and differentiation in the intestinal epithelium.

### Loss of epithelial TFEB exacerbates DSS-induced colitis

**Figure 2:**
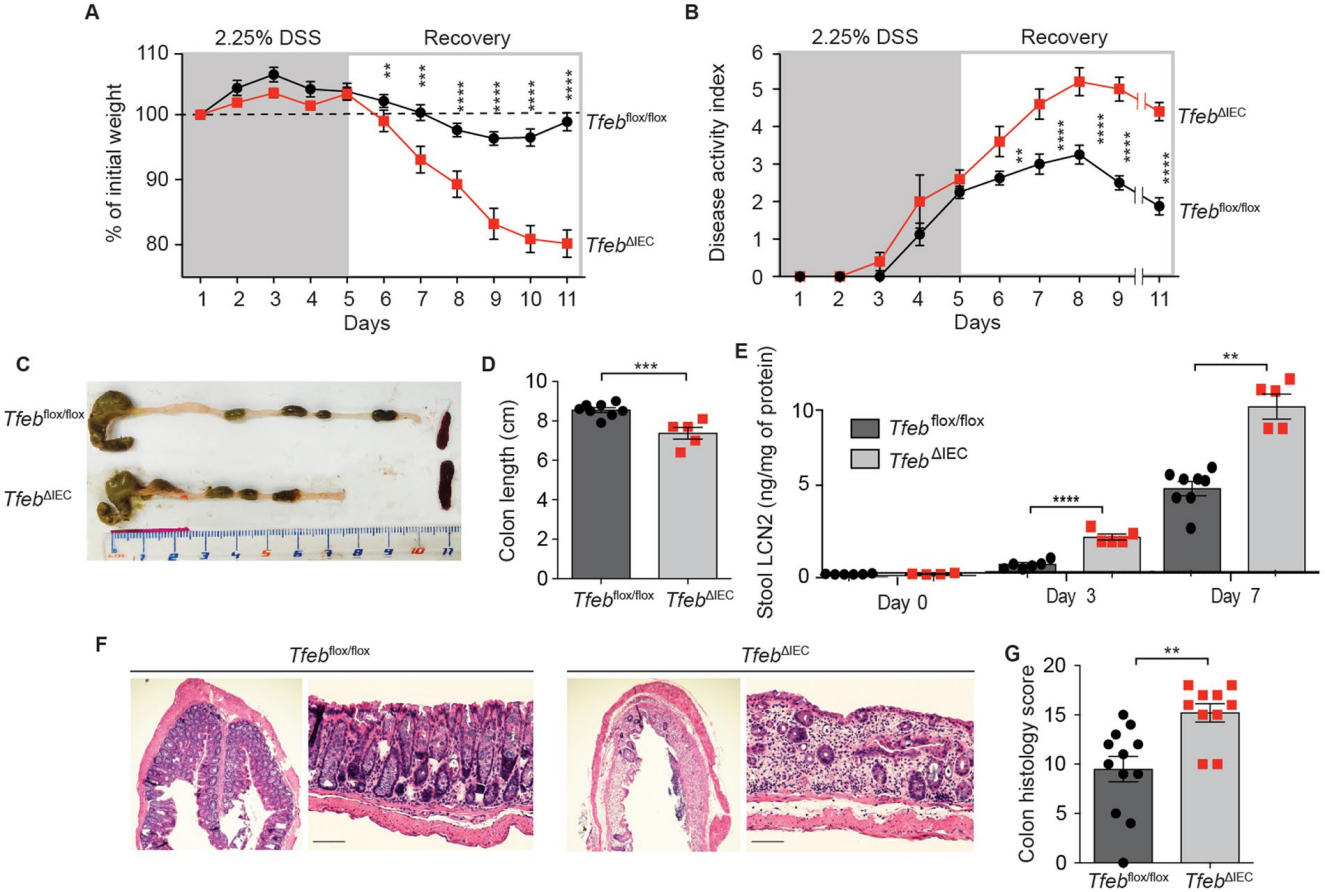
Epithelial deletion of TFEB results in greatly enhanced susceptibility to DSS-induced colitis. (**A**) Body weight and (**B**). Disease activity index as a function of time. *Tfeb* ^*flox/flox*^ mice N = 8, *Tfeb* ^ΔIEC^ mice N = 5. Data are means, error bars are S.E.M. **p < 0.01, ***p < 0.001, ****p < 0.0001 (two-way ANOVA test). Representative of two independent trials. (**C**) Colonic lengths and spleen sizes of representative *Tfeb* ^ΔIEC^ and *Tfeb* ^*flox/flox*^ animals at Day 11. (**D**) Quantification of colon length. *Tfeb* ^*flox/flox*^ mice N = 8, *Tfeb* ^ΔIEC^ mice N = 5. Data are means, error bars are S.E.M. ***p < 0.001 (two-sample *t* test). (**E**) Stool Lipocalin 2 (LCN2) as a function of time. *Tfeb* ^*flox/flox*^ mice N = 8, *Tfeb* ^ΔIEC^ mice N = 5. Data are means, error bars are S.E.M. **p < 0.01, ****p < 0.0001 (two-way ANOVA test). (**F**) H&E staining of colonic sections of representative *Tfeb* ^ΔIEC^ and *Tfeb* ^*flox/flox*^ animals at Day 11. Scale bars represent 50 μm. (**G)** Colon histology score at Day 11. *Tfeb* ^*flox/flox*^ mice N = 12, *Tfeb* ^ΔIEC^ mice N = 10. Data are means, error bars are S.E.M. **p < 0.01 (two-sample *t* test).

To investigate the importance of epithelial TFEB during intestinal distress, we used DSS as a method to perturb homeostasis^22^. In this method, we induced chemical injury to the epithelium in gender-matched littermate mice with low-dose DSS for 5 days, followed by a recovery period of 6 days. We monitored body weight and disease activity index, a quantitative measure of colitis, over the entire time course (see *Methods*). *Tfeb* ^ΔIEC^ mice exhibited dramatically enhanced body weight loss (Fig. 2A) and disease activity index (Fig. 2B), suggesting that DSS treatment caused much greater inflammation in *Tfeb* ^ΔIEC^ animals than in *Tfeb* ^*flox/flox*^ controls. Consistent with this interpretation, the colons of *Tfeb* ^ΔIEC^ animals were much shorter than those from control mice after completion of the time course (Fig. 2C,D). Fecal Lipocalin 2 is a sensitive biomarker for intestinal inflammation in inflammatory bowel diseases^23^ and rapidly increases after DSS treatment in mouse models^24^. We observed a significantly greater increase in fecal Lipocalin 2 in *Tfeb* ^ΔIEC^ animals compared to *Tfeb* ^*flox/flox*^ controls at 3 and 7 days after DSS treatment (Fig. 2E). Furthermore, even after the recovery phase, *Tfeb* ^ΔIEC^ mice exhibited profound epithelial damage in colonic tissue, with a marked decrease in the number of crypts, severe infiltration of immune cells, and a much greater histological pathology score (Fig. 2F,G). These observations confirmed that inflammation after injury in animals that lacked TFEB in the intestinal epithelium was enhanced and persistent, suggesting that TFEB plays a critical role in the protection and/or recovery of intestinal epithelial cells from injury.

### Epithelial TFEB is required for restoration of the epithelial barrier

**Figure 3:**
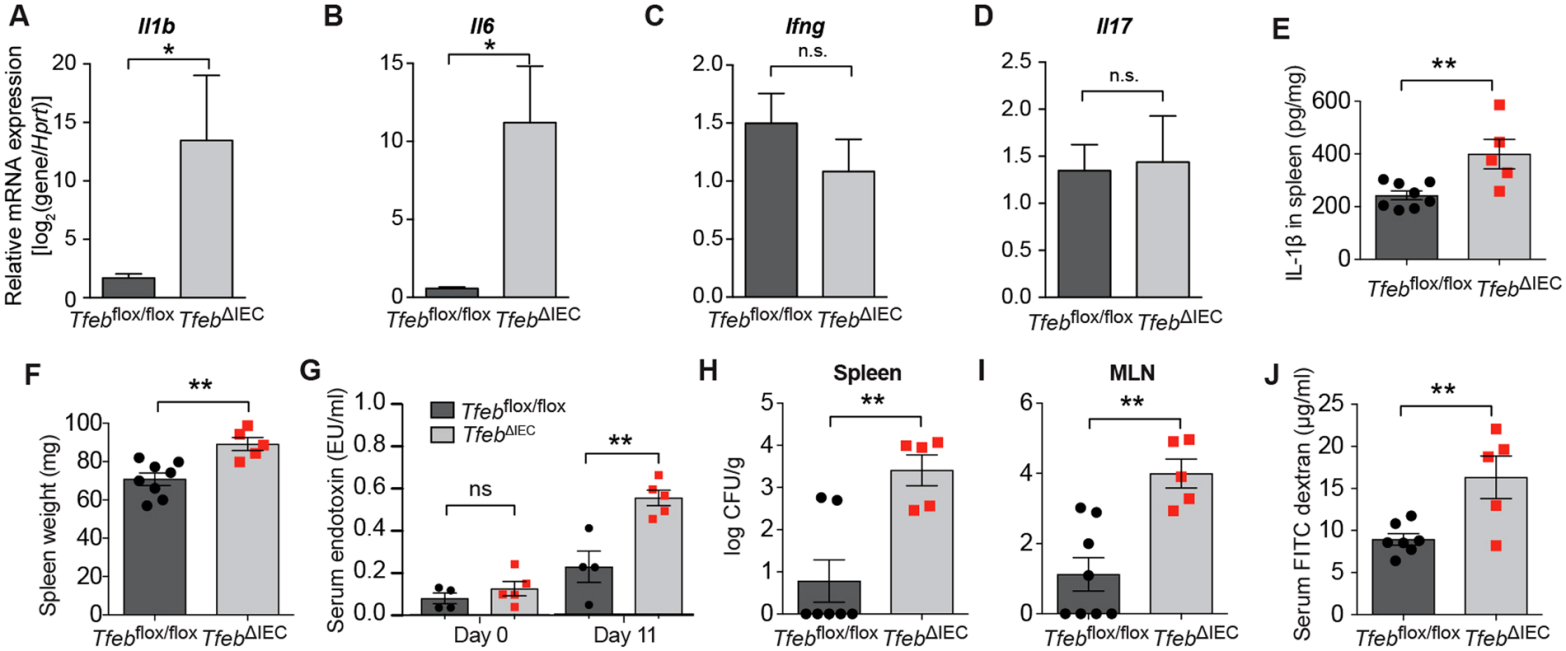
Increased inflammation and bacterial translocation in *Tfeb* ^ΔIEC^ animals. (**A**–**D**) qRT-PCR from colonic tissue of genes *Il1b* (a), *Il6* (b), *Ifng* (c), and *Il17* (d) relative to reference gene *Hprt*. Data are means of three biological replicates, error bars are S.E.M. *p < 0.05 (two-sample *t* test). (**E**) Il-1β ELISA using splenic extracts from *Tfeb* ^ΔIEC^ and *Tfeb* ^*flox/flox*^ animals. *Tfeb* ^*flox/flox*^ mice N = 8, *Tfeb* ^ΔIEC^ mice N = 5. Data are means, error bars are S.E.M. **p < 0.01 (two-sample *t* test). (**F**) Splenic weights from *Tfeb* ^ΔIEC^ and *Tfeb* ^*flox/flox*^ animals. Data are means, error bars are S.E.M. **p < 0.01 (two-sample *t* test). (**G**) Baseline and day 11 serum endotoxin levels. Data are means, error bars are S.E.M. **p < 0.01 (two-sample *t* test). (**H**,**I**) Bacterial colony-forming units (CFU) per g of tissue in spleens (**H**) and mesenteric lymph nodes (**I**). *Tfeb* ^*flox/flox*^ mice N = 7, *Tfeb* ^ΔIEC^ mice N = 5 (**H**). *Tfeb* ^*flox/flox*^ mice N = 8, *Tfeb* ^ΔIEC^ mice N = 5 (**I**). Data are means, error bars are S.E.M. **p < 0.01 (two-sample *t* test). (**J**) Intestinal permeability measured by FITC dextran in serum. *Tfeb* ^*flox/flox*^ mice N = 7, *Tfe*b^ΔIEC^ mice N = 5. Data are means, error bars are S.E.M. **p < 0.01 (two-sample *t* test).

To gain further insight into the consequences of TFEB deletion, we examined the expression of pro-inflammatory cytokines in colon tissue of animals at the end of the recovery phase (day 11). Using qRT-PCR, we observed increased expression of genes *Il1b* and *Il6*, which encode IL-1β and IL-6, respectively, in *Tfeb* ^ΔIEC^ colons (Fig. 3A,B). In contrast, the expression of *Ifng* (IFN-γ) and *Il17* (IL-17) remained unaffected by TFEB status (Fig. 3C,D). These results suggested that *Tfeb* ^ΔIEC^ animals specifically exhibit greater expression of pro-inflammatory mediators IL-1β and IL-6. We also detected higher IL-1β protein levels in spleens of *Tfeb* ^ΔIEC^ animals (Fig. 3E), suggesting that they develop greater systemic as well as local inflammation. Consistent with this idea, *Tfeb* ^ΔIEC^ animals displayed enlarged spleens after recovery from DSS (Fig. 3F). Collectively, these data support a role for TFEB expressed in IECs to limit inflammation, and thus to affect the healing of the intestinal epithelial barrier after injury.

After the recovery phase, we measured bacterial endotoxin in the serum, which provides a quantitative measure of impaired intestinal barrier integrity and translocation of microbial products into the bloodstream. Serum endotoxin levels in untreated *Tfeb* ^ΔIEC^ and *Tfeb* ^*flox/flox*^ animals were not significantly different, indicating the absence of major defects in intestinal permeability at baseline (Fig. 3G). During DSS administration, serum LPS levels increase^22^. As previously shown in wild type mice^25^, serum endotoxin levels returned to normal in *Tfeb* ^*flox/flox*^ control mice after the recovery phase (Fig. 3G). In contrast, *Tfeb* ^ΔIEC^ animals still exhibited three times the normal level of endotoxin during the recovery phase, suggesting that barrier function had not been fully restored (Fig. 3G). Consistent with this result, commensal bacterial translocation to the spleen and the mesenteric lymph nodes remained almost 2 logs higher in the *Tfeb* ^ΔIEC^ animals compared to *Tfeb* ^*flox/flox*^ controls (Fig. 3H,I). These mice also exhibited significantly increased levels of FITC-dextran in the serum after gavage, consistent with a persistent disruption of the intestinal epithelial barrier of *Tfeb* ^ΔIEC^ mice after recovery from DSS treatment (Fig. 3J). These results indicate that epithelial TFEB is critical for barrier restoration after epithelial injury.

### Defective Paneth cells in *Tfeb*^*ΔIEC*^ animals

**Figure 4:**
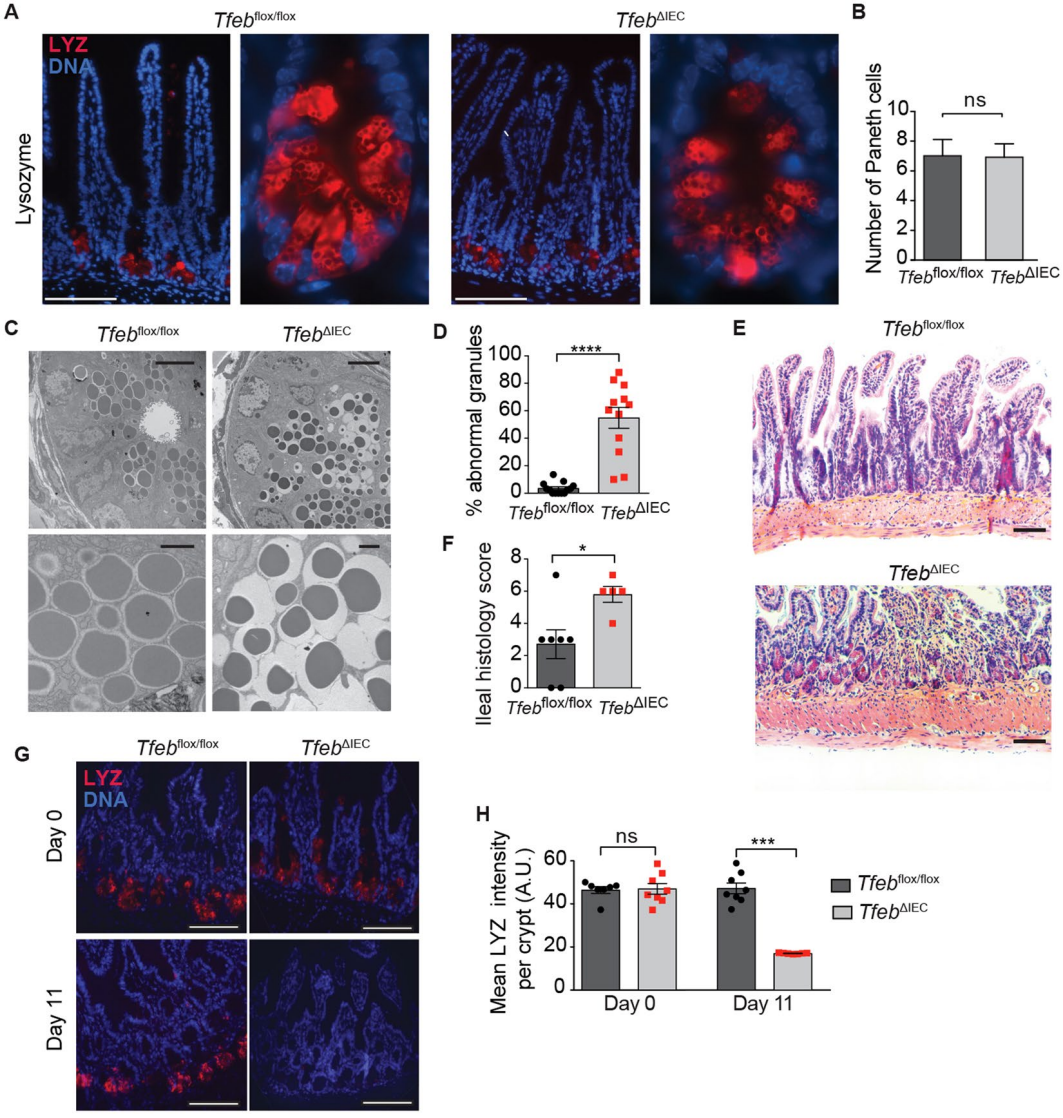
Paneth cell abnormalities in *Tfeb* ^ΔIEC^ animals. (**A**) Lysozyme staining by immunofluorescence in small intestine of representative *Tfeb* ^ΔIEC^ and *Tfeb* ^*flox/flox*^ animals at baseline. Scale bars represent 100 μm. (**B**) Quantification of Paneth cells per crypt. Data are means, error bars are S.E.M. (**C**) Transmission electron microscopy of representative Paneth cells in *Tfeb* ^ΔIEC^ and *Tfeb* ^*flox/flox*^ animals. Scale bars: 5 µm (Top), 1 µm (Bottom). (**D**) Quantification of Paneth cell granule abnormality. N = 12 cells per genotype. Data are means, error bars are S.E.M. (**E**) H&E staining of small intestine sections from representative *Tfeb* ^ΔIEC^ and *Tfeb* ^*flox/flox*^ animals at day 11. Scale bars, 100 µm. (**F**) Ileal histology score at day 11. *Tfeb* ^*flox/flox*^ mice N = 7, *Tfeb* ^ΔIEC^ mice N = 5. Data are means, error bars are S.E.M. (**G**) Lysozyme immunofluorescence in small intestine of representative *Tfeb* ^ΔIEC^ and *Tfeb* ^*flox/flox*^ animals at baseline and Day 11. Scale bars, 100 µm. (**H**) Quantification of lysozyme staining in *Tfeb* ^ΔIEC^ and *Tfeb* ^*flox/flox*^ animals as shown in f. Data are means, error bars are S.E.M. ns, not significant, *p < 0.05, ***p < 0.001, ****p < 0.0001 (two-sample *t* test).

Intestinal immune homeostasis is maintained in part by specialized secretory epithelial cells that reside in the crypts of the small intestine, known as Paneth cells^26^. A major function of Paneth cell is to secrete antimicrobial peptides, including α defensins (or cryptdins), lysozyme, and phospholipase A1. α defensins are mostly expressed in the small intestine^27^, but are functional in the colon^28^. Paneth cell defects and reduced α defensin expression have been linked to inflammatory bowel disease in humans^29–33^ and to enhanced susceptibility to DSS-induced colitis in mice^34,35^. Therefore, we examined the phenotype of Paneth cells in *Tfeb* ^ΔIEC^ animals. Lysozyme immunofluorescence staining revealed no gross differences in Paneth cell number, organization, or morphology between *Tfeb* ^ΔIEC^ and *Tfeb* ^*flox/flox*^ animals at baseline (Fig. 4A,B). In contrast, ultrastructural examination of intracellular secretory granules using transmission electron microscopy revealed a defect in *Tfeb* ^ΔIEC^ Paneth cells. While the granules in *Tfeb* ^*flox/flox*^ Paneth cells presented the expected electron-dense material tightly surrounded by membrane, *Tfeb* ^ΔIEC^ Paneth cells presented abnormal granule morphology, characterized by partial filling of the membrane-bound compartment, suggestive of a granule biogenesis defect (Fig. 4C,D). Similar Paneth cell granule defects had been previously observed in Atg4B-deficient mice^34^, suggesting that deletion of TFEB in IECs may affect a related mechanism at baseline. These results indicate that epithelial expression of TFEB is required for proper assembly of Paneth cell secretory granules.

Based on these findings in Paneth cells, we examined the distal small intestine tissue after recovery from DSS injury. Histologic assessment confirmed more severe histopathology in distal small intestine of *Tfeb* ^ΔIEC^ mice at day 11 (Fig. 4E,F). At baseline and after the recovery phase of DSS colitis, *Tfeb* ^*flox/flox*^ animals had similar numbers of lysozyme+ Paneth cells in the ileum, as evidenced by anti-lysozyme staining. In contrast, *Tfeb* ^ΔIEC^ animals lacked lysozyme+ Paneth cells in the ileum after the recovery phase (Fig. 4G,H), suggesting that epithelial TFEB is essential for Paneth cell survival and restitution after intestinal injury.

### Loss of epithelial TFEB results in minor transcriptional defects at baseline, major changes during injury

**Figure 5:**
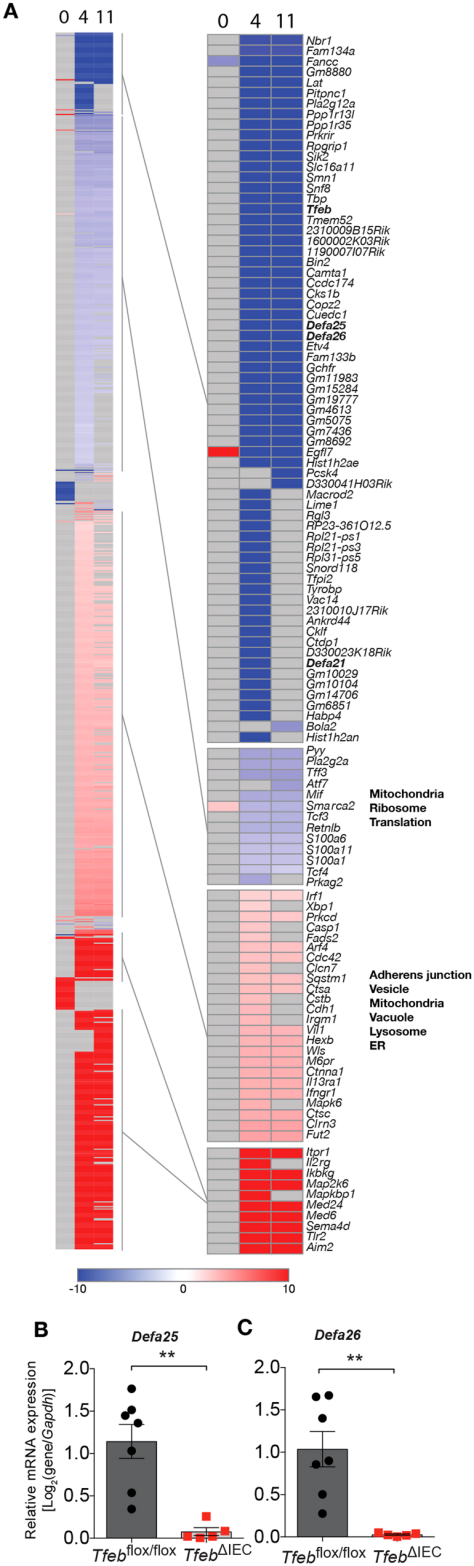
Transcriptional defects in enterocytes from DSS-treated *Tfeb* ^ΔIEC^ animals. (**A**) Differentially expressed genes at days 4 and 11. Over-represented GO annotations are indicated to the right of the respective clusters. (**B**,**C**) qRT-PCR of *Defa25* (c) and *Defa26* (d) relative to reference gene *Gapdh* in *Tfeb* ^ΔIEC^ and *Tfeb* ^*flox/flox*^ animals at day 11. *Tfeb* ^*flox/flox*^ mice N = 7, *Tfeb* ^ΔIEC^ mice N = 5. Data are means, error bars are S.E.M. **p < 0.01, (two-sample *t* test).

To determine the transcriptional effects of loss of TFEB function, we characterized the transcriptomes of colonic epithelial cells from *Tfeb* ^*flox/flox*^ and *Tfeb* ^ΔIEC^ animals by RNA-seq. At the end of the DSS administration (Day 4) and after the recovery period (Day 11), a large set (360 genes) were significantly downregulated in *Tfeb* ^ΔIEC^ animals compared to *Tfeb* ^*flox/flox*^ controls (Fig. 5A, Table S1). The majority of these transcriptomic differences between *Tfeb* ^ΔIEC^ and *Tfeb* ^*flox/flox*^ animals persisted after the recovery phase (Day 11), consistent with our observation of the large recovery defect in the *Tfeb* ^ΔIEC^ animals. Hierarchical clustering analysis segregated the downregulated genes into two main clusters. The first cluster contained 296 genes downregulated in the 4-fold to 500-fold range. Gene ontology annotations related to Mitochondria, Ribosome, and Translation were significantly enriched in this cluster (Table S2). The second cluster contained 64 genes that were not detected in the *Tfeb* ^ΔIEC^ animals. These included α defensin genes *Defa21*, *Defa25*, and *Defa26*, in addition to *Tfeb*. As mentioned, defensins are antimicrobial peptides mostly secreted by Paneth cells that play a critical role in shaping the microbiome. Proper expression of defensins is required for intestinal homeostasis, and their disruption is linked to dysbiosis^36^. qRT-PCR confirmed lower *Defa25* and *Defa26* expression in epithelial cells of *Tfeb* ^ΔIEC^ animals compared to *Tfeb* ^*flox/flox*^ animals at Day 11 after DSS treatment (Fig. 5B,C). These results support the hypothesis that TFEB is required for proper defensin gene expression after epithelial injury, and are consistent with the observed secretory granule defect at baseline and absence of Paneth cells after recovery in *Tfeb* ^ΔIEC^ animals.

Conversely, 564 genes were significantly upregulated in *Tfeb* ^ΔIEC^ animals compared with *Tfeb* ^*flox/flox*^ controls, during DSS induction and recovery (Fig. 5A, Table S1). These segregated into two clusters as well; 217 genes that were not detected in the *Tfeb* ^*flox/flox*^ animals, and 347 that were upregulated in *Tfeb* ^ΔIEC^ animals in the 4 to 500-fold range. In the latter category, we identified genes involved in autophagy (*Irgm1*, *Sqstm1*), in the endoplasmic reticulum unfolded protein response (*Xbp1*), and in lysosomal function (Cathepsin genes *Ctsa*, *Ctsb*, and *Ctsc*, mannose-6-phosphate receptor *M6pr*, and β-hexosaminidase subunit β *Hexb*). Only the latter cluster exhibited enrichment of gene ontology annotation categories, which were related to adherens junctions, vesicle/vacuole/lysosome, mitochondria, and the endoplasmic reticulum (Table S2). Taken together, these findings surprisingly showed no defect in the expression of lysosomal and autophagy genes in *Tfeb* ^ΔIEC^ animals, and suggested that unknown cellular mechanisms may be activated to compensate for the loss of TFEB within intestinal epithelial cells. These putative compensatory mechanisms may induce the expression of innate immunity, cell adhesion, and organelle genes in an attempt to restore tissue homeostasis. Alternatively, the observed gene induction could be secondary to the enhanced inflammatory infiltration caused by loss of TFEB.

**Figure 6:**
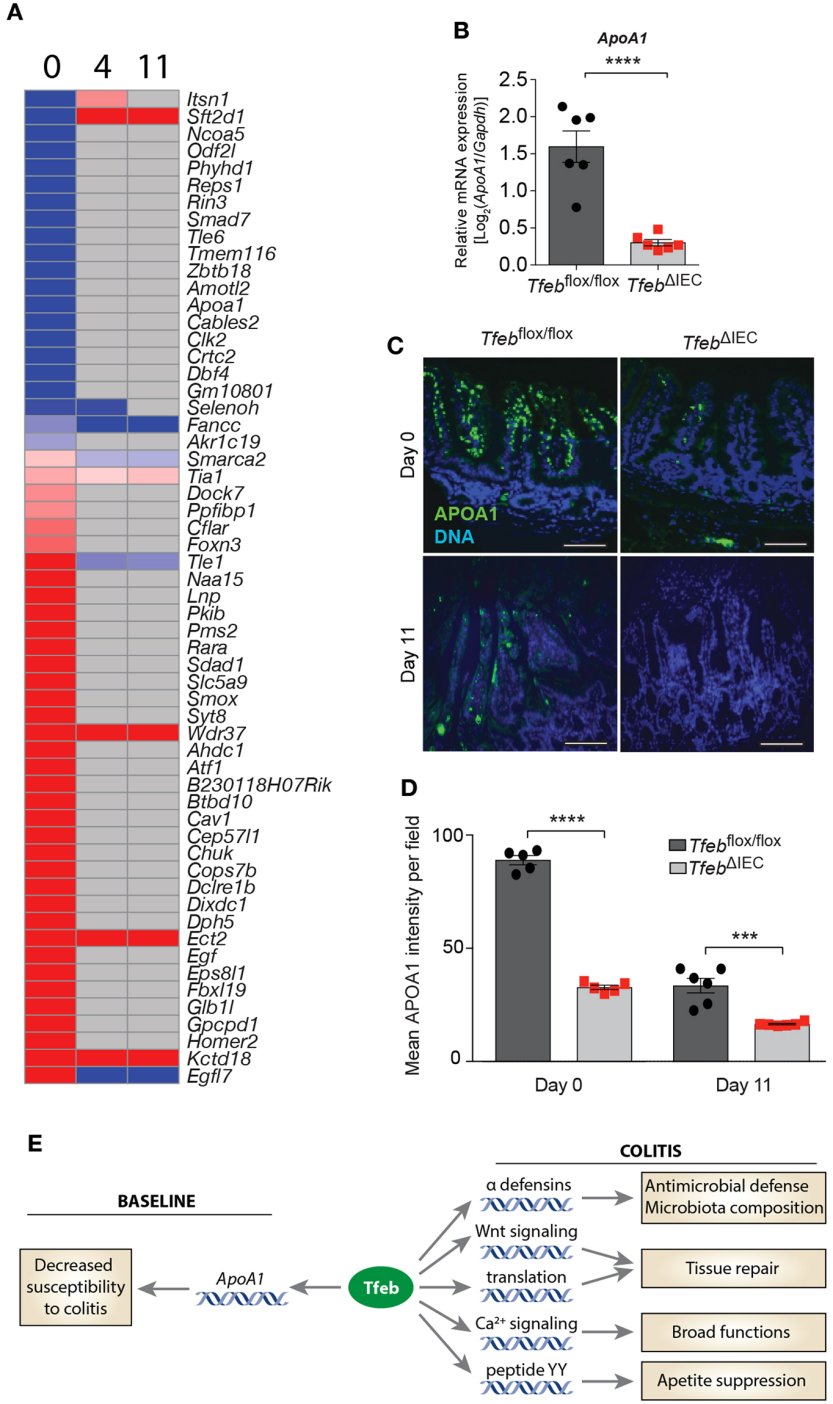
Transcriptional defects in enterocytes from unperturbed *Tfeb* ^ΔIEC^ animals. (**A**) Differentially expressed genes at day 0 (untreated), comparing *Tfeb* ^ΔIEC^ to *Tfeb* ^*flox/flox*^ animals as reference. (**B**) qRT-PCR of *ApoA1* relative to reference gene *Gapdh* in *Tfeb* ^ΔIEC^ and *Tfeb* ^*flox/flox*^ animals at baseline. *Tfeb* ^*flox/flox*^ mice N = 6, *Tfeb* ^ΔIEC^ mice N = 6. ****p < 0.0001 (two-sample *t* test). (**C**) Anti-APOA1 immunofluorescence in representative *Tfeb* ^ΔIEC^ and *Tfeb* ^*flox/flox*^ animals at baseline and day 11. Scale bars, 100 µm. (**D**) Quantification of anti-APOA1 immunofluorescence. ***p < 0.001, ****p < 0.0001 (two-way ANOVA test). (**E**) Working model for the observed TFEB-dependent decreased transcription of genes at baseline (left) and during colitis (right). The arrows connecting TFEB to each gene/category are not meant to imply direct regulation.

In contrast to these large differences during and after DSS treatment, there were few differentially expressed genes between unperturbed *Tfeb* ^*flox/flox*^ and *Tfeb* ^ΔIEC^ animals (Fig. 6A, Table S1). Just 21 genes were significantly downregulated in *Tfeb* ^ΔIEC^ animals compared to *Tfeb* ^*flox/flox*^ controls, yet none of these were known autophagy or lysosomal biogenesis genes. This suggested the unexpected conclusion that TFEB is dispensable in the colonic epithelium for autophagy and lysosomal gene expression at baseline.

*ApoA1* encodes apolipoprotein A1, which is the major constituent of high density lipoprotein (HDL)^37^. APOA1 in HDL is important for the transport of cholesterol and phospholipids from the periphery to the liver. Importantly, deficiency in human *APOA1* expression has been linked to susceptibility to inflammatory bowel disease in humans^38,39^. Furthermore, loss of intestinal *ApoA1* results in greatly enhanced susceptibility to DSS-induced colitis and malignant transformation in mice^40^. In fact, overexpression of human APOA1 protects mice from DSS colitis^41^. These previous studies establish causality between APOA1 expression and intestinal inflammation. Strikingly, *ApoA1* was strongly downregulated in *Tfeb* ^ΔIEC^ animals (Fig. 6A), suggesting that APOA1 could contribute to TFEB-mediated epithelial protection.

### Defective APOA1 expression in *Tfeb*^ΔIEC^ animals

Using qRT-PCR, we confirmed that intestinal epithelial cells from unperturbed *Tfeb* ^ΔIEC^ animals express much lower *ApoA1* mRNA than *Tfeb* ^*flox/flox*^ animals (Fig. 6B). Immunofluorescence staining for APOA1 revealed a major reduction in epithelial APOA1 in unperturbed animals (Fig. 6C,D). After recovery from DSS colitis, while *Tfeb* ^*flox/flox*^ animals exhibited reduced but detectable APOA1 expression, APOA1 expression in *Tfeb* ^ΔIEC^ animals was lost (Fig. 6C,D). These results demonstrate a severe reduction in APOA1 expression caused by TFEB deficiency in the intestinal epithelium at baseline and after recovery from injury. Such defects in APOA1 expression could provide a mechanistic basis for the greatly enhanced susceptibility of *Tfeb* ^ΔIEC^ animals to chemically-induced colitis.

## Discussion

We report here the first functional analysis of TFEB in the intestinal epithelium, and identify TFEB as a critical determinant of susceptibility to epithelial injury. We found that TFEB is constitutively expressed in intestinal epithelial cells in healthy animals. Conditional deletion of TFEB in the intestinal epithelium resulted in alterations to the Paneth cell compartment of the small intestine, and in minor transcriptional changes in colonic epithelial cells at baseline. Of note, *ApoA1* expression was significantly downregulated at baseline in *Tfeb* ^ΔIEC^ animals (Fig. 5H). The lack of a major transcriptional effect of TFEB deletion on the expression of genes in intestinal epithelial cells of unperturbed animals suggests that TFEB is not strongly engaged under baseline homeostatic conditions. This is consistent with our finding that TFEB localizes primarily to the cytosol of the intestinal epithelial cells in healthy animals, because cytosolic retention of TFEB is a major mode of its regulation^42^.

Animals lacking TFEB in the intestinal epithelium were much more susceptible to intestinal injury compared to control animals. Conditional deletion of epithelial TFEB resulted in greatly enhanced pathology throughout the entire intestine after DSS treatment, as measured by several parameters, including cytokine expression, body weight loss, disease severity, and markers of inflammation in stool. The epithelial barrier exhibited more severe breakdown, as evidenced by increased permeability and bacterial translocation to distal organs. During the recovery phase, wild type mice were able to recover and repair the damage to the epithelium while conditionally deleted animals were unable to do so. These observations reveal a key role for TFEB deletion in the ability to protect the epithelium from chemical injury, and to repair the damage caused by the resulting exuberant inflammatory response.

Transcriptional profiling revealed a profound effect of TFEB deletion on the transcriptome of colonic enterocytes in DSS-treated animals (Fig. 6E). Among the downregulated genes, the notable absence of autophagy or lysosomal genes indicates that TFEB is not required for their expression in the intestinal epithelium, which is in stark contrast to other cell types, including HeLa cells and hepatocytes^16^. In contrast, the α defensin genes *Defa25* and *Defa26* were strongly dependent on TFEB. Defensins are antimicrobial peptides expressed by the intestinal epithelium, and are known to play important roles in intestinal homeostasis and host defense against infection^43^. Our results suggested that Tfeb is required for the expression of a subset of α defensin genes. These results are consistent with the striking defect in Paneth cell secretory granule morphology, and collectively suggest an important role for Tfeb in secretory cells of the ileum and colon.

Interestingly, the expression of trefoil-factor 3 (Tff3), which is important for intestinal tissue repair^44^, and of Peptide YY, which is expressed by enteroendocrine cells and mediates communication with the central nervous system to induce satiety^45^, were also reduced in the knockout. For the most part, genes that participate in autophagy or lysosomal biogenesis appeared to be unaffected, which could be a result of the action of the TFEB-related transcription factor Tfe3^46^. Whether Tfe3 is capable of compensating for the loss of TFEB in the intestinal epithelium for the expression of autophagy and lysosomal genes is an important question that requires further examination. Overall, these results show that TFEB is required in the intestine for the expression of genes that participate in antimicrobial defense, tissue repair, and appetite control.

The loss of APOA1 expression that we observed in unperturbed *Tfeb* ^ΔIEC^ mice could explain, at least partially, their enhanced susceptibility to epithelial injury and colitis. In humans, loss of APOA1 causes familial HDL deficiency, Tangier disease, and familial visceral amyloidosis^47^. APOA1 expression is reduced in Crohn’s disease patients, and APOA1 levels inversely correlate with disease activity^38,48^. Furthermore, APOA1 transcription is reduced in the ileal intestinal epithelium of pediatric CD patients, and is part of a specific transcriptional signature for that population^38,39^. Recent studies showed that APOA1-deficient mice exhibit greatly enhanced susceptibility to DSS colitis and colitis associated carcinoma, establishing a causal link between APOA1 status and intestinal inflammation in mammals^40^. Furthermore, overexpression of human APOA1 is sufficient to protect mice from DSS colitis^41^. The exact molecular mechanism by which APOA1 protects against epithelial injury and malignancy is unknown. One proposed mechanism involves sequestration of LPS by HDL, thus reducing pro-inflammatory signaling through TLR4^49^. Because *Tfeb* ^ΔIEC^ mice express greatly reduced levels of APOA1 in their intestinal epithelium, it is possible that their lack of APOA1 results in higher levels of LPS leakage during the initial stages of DSS induction, resulting in more severe inflammation and non-resolving tissue damage. Thus, we document a novel role for TFEB in the expression of APOA1, a known protective factor against epithelial injury and colitis in rodents and humans.

The importance of TFEB and related transcription factors in inflammation and immunity is emerging. Both TFEB and TFE3 were shown to be activated by LPS in macrophages, and to directly trigger a sizable portion of the downstream transcriptional response^46^. Among the genes that are affected by TFEB and TFE3 deletion are IL-6 and TNFα, which are major contributors to intestinal inflammation *in vivo* ^11,46^. In addition, TFEB was shown to be activated in macrophages during Fc receptor-mediated phagocytosis, and in dendritic cells during treatment with LPS^50,51^. In this context, TFEB is important for antigen cross-presentation with important consequences for the adaptive immune response. Finally, TFEB was shown to be involved in a lysosomal homeostatic mechanism mediated by Cathepsin B during *F*. *novicida* infection in macrophages^52^. It is important to determine the contribution of TFEB to immune homeostasis and host defense against diverse challenges, including epithelial injury, *in vivo*. From the present study, it is apparent that TFEB exerts an important function in intestinal epithelial cells to ensure proper Paneth cell function and protect the intestinal epithelium from injury.

## Experimental Procedures

### Mice

Mice were bred in specific-pathogen-free facilities at Massachusetts General Hospital. All experiments were conducted following protocols approved by the animal ethics committees at Massachusetts General Hospital. Mice expressing cre recombinase under control of the villin promoter (*Villin-cre*) were purchased from Jackson Laboratory. *Tfeb* ^*flox/flox*^ mice^18^ were obtained from Telethon Institute of Genetic Medicine in Naples, Italy. These mice were bred with *Villin-cre* mice to create mice with conditional targeted deletion of *Tfeb* gene. All the mice were 8–12 weeks old at the time of all the experiments.

### Tissue preparation for immunofluorescence

Mice were sacrificed by CO_2_ asphyxiation. Pieces of small intestine and colon were quickly dissected, opened longitudinally and washed in PBS. 4% paraformaldehyde (WAKO) was used for fixation. Fixed tissues were immersed in PBS containing 30% sucrose (Sigma-Aldrich) for overnight, embedded in OCT compound (Sakura Finetek USA) and frozen at −80 °C. 8 μm sections were cut using Leica CM3050 cryostat (Leica Microsystems) and mounted on microscope slides.

### Immunofluorescence staining

Sections were air-dried and microwaved for 10 min in 10 mM citrate buffer. 10% donkey serum was used for blocking during staining. Sections were then incubated with diluted primary antibody as described below for 18 hours at 4 °C. Slides were washed 3 times in PBS for 5 min and then incubated with diluted secondary antibody for 1 h at room temperature. Slides were washed in PBS again and mounted in Vectashield medium with DAPI (Vector Laboratories). The primary antibodies used were rabbit anti-TFEB (1:250, Bethyl Laboratories), mouse anti-Ki-67 (1:1000, EBIOSCIENCE), rabbit anti-lysozyme (1:1000, DACO) and rabbit anti-apolipoprotein A1 Antibody (1 ug/ml, Invitrogen). The secondary antibodies used were donkey anti-Rabbit Alexa488 (1:200, Jackson Immunoresearch), donkey anti-Mouse Alexa488 (1:200, Jackson Immunoresearch), and donkey anti-Rabbit Alexa 594 (1:200, Jackson Immunoresearch). Images were collected using a confocal fluorescent microscope (Nikon, Melville, NY).

### IEC isolation

IEC isolation was performed as previously described^31^. Briefly, ileum and colon were removed and separated, opened longitudinally, washed with PBS and further minced into pieces. Tissues were treated with 1 mM DTT in PBS for 10 min and further treated with 30 mM EDTA (Boston BioProduct) in PBS for 30 min at room temperature. Isolated IECs were collected by centrifuge and used for Western blot or RNA analysis.

### HE/PAS staining

A piece of small intestine and colon were harvested and quickly fixed in 10% formalin overnight at 4 °C. Fixed tissues were embedded in paraffin and 5 μm section was stained with Hematoxylin/Eosin (HE) or Periodic acid Schiff (PAS). The number of goblet cells was quantified by counting PAS-positive cells of at least 20 villi from 3 individual mice.

### Quantification of Immunofluorescence staining

Number of positive staining cells was counted by analyzing randomly selected 20 crypts from at least 3 independent mice. Analyzer was blinded to the origin of tissues. Fluorescence intensity measurement was done using ImageJ software (NIH) thresholding tool in region of interest with the help of ROI Manager. Staining and image acquisition performed in parallel for the entire set with identical image acquisition settings and exposure times.

### Electron microscopy

Area of electron-dense core (ED) and an electron-lucent peripheral halo (EL) in each secretory granule was measured using ImageJ software (NIH). The percentage of granules with EL/ED + EL ≥ 40% was reported as abnormal granule percentage.

### DSS treatment

Littermates with gender matched controls were used for all DSS experiments. Mice were fed 2.25% DSS (MW = 40000) dissolved in sterile water for 5 consecutive days, followed by being fed with sterile water for 6 additional consecutive days. Body weight of each mouse was measured daily. Disease activity indices were measured daily. The following scoring system was used for the daily activity index: Stool consistency: 0; normal stool, 1; soft stool, 2; very soft stool, 3; diarrhea, Stool blood content: 0; negative hemoccult: 1; positive hemoccult: 2; traces of blood: 3; visible rectal bleeding. Stool consistency and blood content scores were then combined. On day 11 after initial treatment of DSS, mice were sacrificed and colon length was measured. Distal colon was dissected for HE staining and RNA isolation. Colon histology was scored based on scoring system as previously described^22^. Briefly, severity of inflammation (0–3), depth of injury (0–3), and crypt damage (0–4) were estimated, then total scores were multiplied by a factor representing the percentage of tissue involvement: ×1 (0–25%), ×2 (26–50%), ×3 (51–75%), ×4 (76–100%).

### Western blot analysis

Cells were lysed in RIPA buffer (Boston BioProduct) with Complete protease inhibitor (Roche) and Phosphatase inhibitor cocktail (Cell Signaling) for 15 min on ice. Lysates were pelleted and used for western blotting. The antibodies used were rabbit anti-TFEB (1:1000, Bethyl Laboratories), mouse anti-β-actin (1:5000, SIGMA).

### Real-time Quantitative PCR

RNA isolation was performed using the RNeasy kit (Qiagen). cDNA was synthesized using iScript cDNA Synthesis Kit (BioRad). Quantitative PCR reaction was run using the iQ SYBR Green Supermix (BioRad) and the CFX384 Touch Real-Time PCR Detection System (BioRad). Relative quantification was performed by normalizing to the expression of reference genes *Hprt* or *Gapdh*. Primers used were as follows: mouse *Tfeb* Fwd: TGAGATGCAGATGCCTAACACGCT, Rev: TTGTCTTTCTTCTGCCGCTCCTTG, mouse *Il1b* Fwd: ACCTCACAAGCAGAGCACAA, Rev: TTGGCCGAGGACTAAGGAGT, mouse *Il6* Fwd: TAGCTATGGTACTCCAGAAGAC, Rev: ACGATGATGCACTTGCAGAA, mouse *Ifng* Fwd: ATGAACGCTACACACTGCATC, Rev: CCATCCTTTTGCCAGTTCCTC, mouse *Il17* Fwd: TTTAACTCCCTTGGCGCAAAA, Rev: CTTTCCCTCCGCATTGACAC, mouse *Hprt* Fwd: AGTCCCAGCGTCGTGATTAG, Rev: TGATGGCCTCCCATCTCCTT, mouse *Gapdh* Fwd: CATCACTGCCACCCAGAAGACTG, Rev: ATGCCAGTGAGCTTCCCGTTCAG. *Apoa1* Fwd: GGCAGAGACTATGTGTCCCAGT, Rev: GCTGACTAACGGTTGAACCCAG. *Defa25* Fwd: GTGAAGATCTGATATGCTATTG, Rev: ACCAGAGCATGTACATTAAATG. *Defa26* Fwd: TACTGAGGTGCAGCCACAGGAA, Rev: GCCTCTTTTTCTACAATAGCATCC.

### RNAseq

Intestinal epithelial cells were isolated from the colons of 8–12 week old mice at 0, 4, and 11 days post-DSS treatment. For each timepoint, 2 *Tfeb* ^ΔIEC^ and 2 *Tfeb* ^*flox/flox*^ animals were used as biological replicates. RNA was extracted by tissuelyzer treatment followed by RNeasy (Qiagen). Full length cDNA libraries were prepared using template switching and whole transcriptome amplification in a slight variation of the SmartSeq. 2 protocol described in^53^. Libraries were sequenced on a MiSeq machine (Illumina). Paired-end reads were mapped to the mm10 reference genome using Tophat2+ Bowtie. Differential gene expression was determined using Cuffdiff^54^. A corrected p-value (q-value) of 0.05 was considered significant. Mapped reads were deposited in GEO with accession number GSE98266. Clustering analysis of differentially expressed genes was performed using Pearson correlation implemented in Morpheus (software.broadinstitute.org/morpheus). GO enrichment analysis was performed for each gene cluster using g:profiler^55^.

### Measurement of inflammation markers by ELISA and LAL assay

Spleen was homogenized in homogenizing buffer (100 mM Tris, pH 7.4, 150 mM NaCl, 1 mM EGTA, 1 mM EDTA, 1% Triton X-100, 0.5% Sodium deoxycholate), the homogenate was centrifuged and supernatants were used for cytokine measurements using mouse IL-1β ELISA kit (eBioscience, 88-7013-22) according to the manufacturer’s protocols, and read on a Luminex machine. In order to assess intestinal inflammation, fecal samples from day 1, 4, and 7 post-DSS treatment were homogenized in homogenizing buffer, spun down and supernatants were collected and assayed for Lipocalin-2 by ELISA according to manufacturer’s instructions (R&D, MLCN20). Serum endotoxin level was measured using ToxinSensor Chromogenic Endotoxin Assay Kit (GenScript, L00350). Briefly, diluted plasma was incubated with Limulus amoebocyte lysate (LAL) and after performing several reactions under endotoxin free condition; samples were read spectrophotometrically at 545 nm. The plasma endotoxin levels were calculated against a standard curve of endotoxin (*E*. *coli* 0113:H10) concentrations of 0.1, 0.05, 0.025, 0.0125 and 0 EU/ml.

### Measurement of CFU in organs

Mesenteric lymph nodes (MLNs) and spleen were aseptically retrieved from mice at day 11. Tissue was homogenized in sterile PBS and plated onto LB agar plates after serial dilution, then incubated at 37 °C for 24 h before colony forming unit (CFU) quantification.

### FITC dextran permeability

Mice were given FITC-dextran tracer (Sigma, 4 kDa, 0.6 mg/g body weight in 0.2 mL PBS) via *intragastric administration* using a ball tip needle and FITC levels were measured 4 h later in hemolysis-free serum using a Fluorescence Spectrophotometer.

## Electronic supplementary material

Supplementary information

Supplementary Dataset 1

Supplementary Dataset 2
