# Supplementary material for: Transcription factor TFEB cell-autonomously modulates susceptibility to intestinal epithelial cell injury in vivo
Source: Sci Rep. 2017 Oct 24;7:13938. doi: 10.1038/s41598-017-14370-4 (PMC5655326; doi:10.1038/s41598-017-14370-4)
Supplement: Supplementary file 1 — Supplementary information [file 41598_2017_14370_MOESM1_ESM.pdf]

Transcription factor Tfeb cell-autonomously modulates susceptibility to intestinal epithelial cell injury *in vivo*

Authors: Tatsuro Murano, Mehran Najibi, Geraldine L.C. Paulus, Fatemeh Adiliaghdam, Aida Valencia-Guerrero, Martin Selig, Xiaofei Wang, Kate Jeffrey, Ramnik J. Xavier, Kara G. Lassen, Javier E. Irazoqui

Supplementary Text.

Figure S1. Tfeb is mainly expressed in intestinal epithelial cells. qRT-PCR of *Tfeb* mRNA, relative to reference gene *Hprt,* in total RNA extracted from whole tissue (*Left*) or from isolated intestinal epithelial cells (*Right*) obtained from $Tfeb^{\Delta IEC}$ and $Tfeb^{flox/flox}$ animals, as indicated. Values are means of 4 $Tfeb^{\Delta IEC}$ and 4 $Tfeb^{flox/flox}$ animals, normalized to the $Tfeb^{flox/flox}$ whole tissue, error bars are S.E.M.

Figure S2. Uncropped images of Western blots. A. Anti-TFEB immunoblot. **B.** Anti-actin immunoblot. Boxes highlight regions used for Fig. 1B.

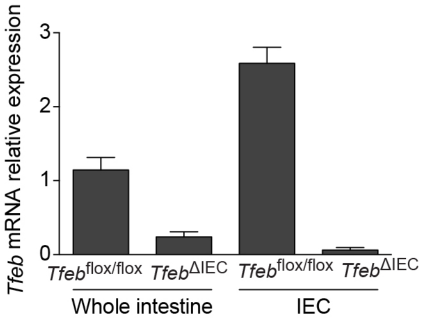

Tfeb mRNA relative expression
3
2
1
0
Tfeb flox/flox
Tfeb ΔIEC
Tfeb flox/flox
Tfeb ΔIEC
Whole intestine
IEC

A

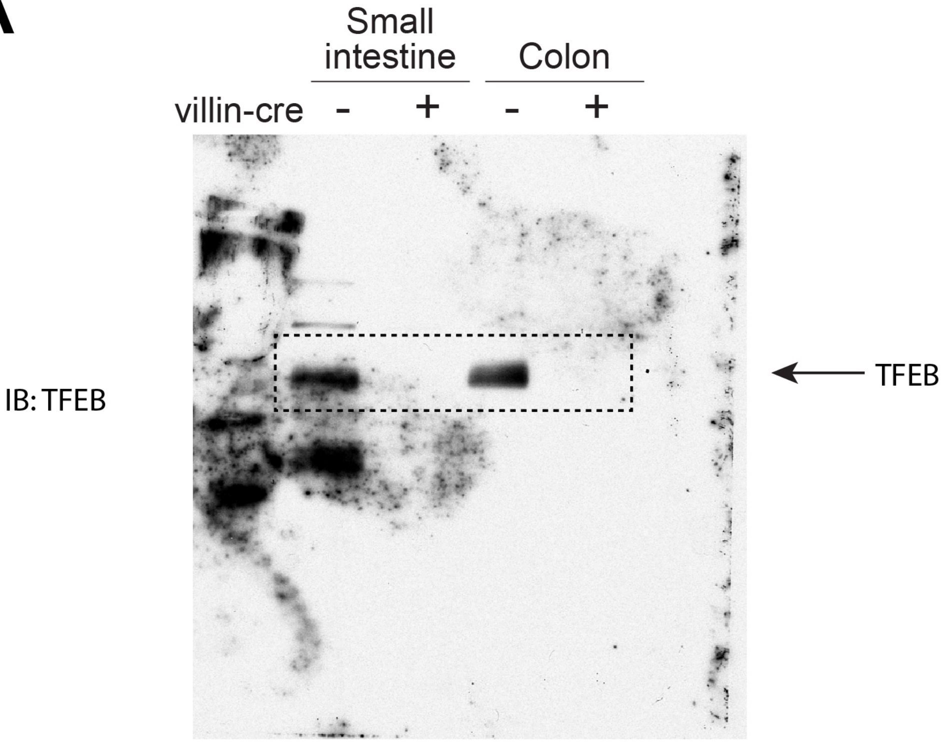
Small intestine
Colon
villin-cre - + - +
IB: TFEB
TFEB

B

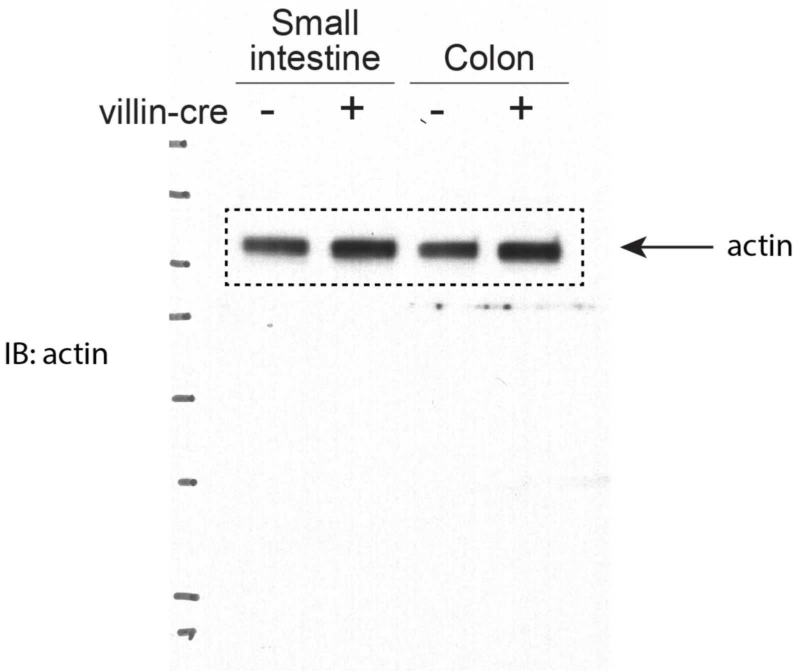
Small intestine
Colon
villin-cre - + - +
IB: actin
actin
